# Whole-Genome Sequencing for Antimicrobial Resistance Detection: A Simulation-Based Evaluation of WHO-Recommended Workflow in Pakistan

**DOI:** 10.12669/pjms.42.4.14515

**Published:** 2026-04

**Authors:** Hayam A. Alrasheed, Asif Jan, Abdur Rahim, Bushra Waheed, Rani Akbar

**Affiliations:** 1 Hayam A. Alrasheed Department of Pharmacy Practice, College of Pharmacy, Princess Nourah bint Abdulrahman University, Saudi Arabia; 2 Asif Jan Saidu Group of Teaching Hospital Saidu Sharif Swat, KP, Pakistan. Department of Pharmacy, University of Peshawar, Peshawar, KP, Pakistan. District Headquarter Hospital Charsadda, Charsadda, KP, Pakistan; 3 Abdur Rahim Department of Pharmacy, Cecos University of IT & Emerging Sciences, Peshawar, KP, Pakistan; 4 Bushra Waheed Department of Pharmacy, University of Swabi, Swabi, KP, Pakistan; 5 Rani Akbar Department of Pharmacy, Abdul Wali Khan University, Mardan, KP, Pakistan

**Keywords:** AMR genes, Antimicrobial resistance, Genomic surveillance, Pakistan, Whole-genome sequencing, WHO

## Abstract

**Objectives:**

To evaluate the performance and feasibility of whole-genome sequencing works for detecting antimicrobial resistance and to determine whether it is practical to use in Pakistan, using a simulation-based model following World Health Organization’s antimicrobial resistance genome sequencing guidelines.

**Methodology:**

A simulation-based evaluation was conducted at the Centre of Genomics, Rehman Medical Institute (January to December 2025) to investigate the effectiveness of whole-genome sequencing (WGS) in detecting antimicrobial resistance (AMR) patterns in Pakistan. A simulation model was developed according to the guidelines described in World Health Organization (WHO) report for detection of Antimicrobial Resistance using Whole Genome Sequencing (WGS). Simulation inputs included common AMR genes circulating in Pakistan (mecA, blaCTX-M, blaNDM-1, blaOXA-48, aac(6’)-Ib), pathogenic species profiles, and metadata recommendations. Evidence from WHO-recommended WGS workflows was used for AMR detection and validation. The study was carried out over a period of 12 months.

**Results:**

Simulated WGS workflows showed high predicted accuracy for AMR gene detection (94–100%), virulence factor identification (≥90%), plasmid and mobile genetic element (MGE) resolution, and outbreak-level genomic typing. WGS reduced theoretical AMR detection time from 48–72 hours (phenotypic methods) to <12 hours post-culture. WHO-recommended quality metrics including ≥30× coverage, Q30 base accuracy, appropriate metadata capture, and standardized laboratory workflows were achievable within Pakistan health care system, though limited by workforce training, bioinformatics infrastructure.

**Conclusion:**

WGS is a feasible and highly accurate method for detecting antimicrobial resistance (AMR) in Pakistan. Using WGS according to the World Health Organization (WHO) recommended standards can greatly improve Pakistan’s national AMR surveillance system.

## INTRODUCTION

Antimicrobial resistance (AMR) represents one of the most pressing global public health threats of the 21st century, with an estimated 4.95 million deaths associated with AMR in 2019 alone, including 1.27 million directly attributable to resistant infections.^1^ The burden of AMR is disproportionately higher in low- and middle-income countries (LMICs), where limited healthcare infrastructure, high rates of infectious diseases, and widespread over-the-counter antibiotic use accelerate the emergence and spread of resistant pathogens.^2^ In Pakistan, increasing reports of multidrug-resistant (MDR) *Klebsiella pneumoniae*, extensively drug-resistant (XDR) *Acinetobacter baumannii*, methicillin-resistant *Staphylococcus aureus* (MRSA), and carbapenemase-producing Enterobacterales, particularly harboring *blaNDM-1* and *blaOXA-48*, highlight a growing public health crisis.^3^-^5^

Conventional diagnostic approaches in Pakistan rely primarily on culture-based antimicrobial susceptibility testing (AST), which typically requires 48–72 hours to yield results and may fail to detect plasmid-borne resistance genes, emerging point mutations, and mobile genetic elements (MGEs) that contribute to AMR dissemination.^6^ Such limitations hinder timely infection control measures and appropriate antimicrobial therapy, often resulting in prolonged hospital stays, increased morbidity, and higher healthcare costs.^7^

Whole-genome sequencing (WGS) has emerged as a transformative technology for AMR surveillance, providing comprehensive insights into the resistome, virulome, plasmids, MGEs, and clonal lineages of bacterial pathogens with high sensitivity and specificity.^8^-^10^ The World Health Organization (WHO) has recognized the utility of WGS in AMR surveillance, releasing the AMR Genome Sequencing Technical Report, which details recommended laboratory workflows, quality control (QC) measures, metadata standards, and best practices for global implementation.^11^ WGS not only outperforms phenotypic AST in detecting low-frequency variants and novel resistance determinants but also enables high-resolution tracking of pathogen transmission, facilitating outbreak investigations and national AMR 5 surveillance strategies.^12^,^13^

Recent studies have validated the concordance between WGS-predicted resistance profiles and phenotypic susceptibility. For instance, Cipriani et al. (2025) demonstrated near-perfect detection of AMR genes, virulence loci, and MGEs in MRSA and ESBL-producing *K. pneumoniae* using optimized WGS workflows.^14^ Similar global studies have confirmed the reliability of WGS in diverse clinical and public health settings, reinforcing its role as a central tool for genomic epidemiology and resistance monitoring.^15^,^16^

Despite these advances, Pakistan currently lacks a coordinated genomic surveillance system for AMR. National reporting relies largely on the WHO Global Antimicrobial Resistance Surveillance System (GLASS), with limited integration of genomic data into routine public health workflows.^17^ Barriers include shortage of trained bioinformaticians, inconsistent power and internet infrastructure, challenges in importing reagents, and insufficient laboratory capacity. Nevertheless, tertiary-care hospitals and provincial health laboratories possess foundational resources that could support the implementation of WGS-based AMR workflows.

Given this context, the present study aimed to develop a simulation-based evaluation of WHO-recommended WGS workflows for AMR detection, using realistic genomic parameters and pathogen epidemiology reflective of Pakistan. By integrating international evidence and WHO guidelines, this analysis seeks to provide actionable insights for establishing WGS-based surveillance frameworks to combat AMR in the country.

## METHODOLOGY

A simulation-based evaluation was conducted at Centre of Genomic Rehman Medical Institute (RMI) Peshawar to assess the performance of whole-genome sequencing (WGS) for antimicrobial resistance (AMR) detection in Pakistan.

### Ethical Approval:

It was obtained from the institutional review board of RMI, Peshawar. Reference (Approval No: 2871/RMI/Pesh; Dated: February 28, 2025)

The study incorporated WHO AMR Genome Sequencing Technical Report recommendations,^11^ global WGS-AMR validation studies^1^ and Pakistan’s national AMR profiles derived from NIH-GLASS and published reports.^18^ The simulation modeled the analytical performance of WGS for AMR detection rather than evaluating sequencing platforms directly. The study was carried out over a period of 12 months (January to December 2025). Bacterial pathogens with the highest prevalence of AMR in Pakistan were included, encompassing methicillin-resistant *Staphylococcus aureus* (MRSA), extended-spectrum beta-lactamase (ESBL)-producing *Klebsiella pneumoniae*, carbapenem-resistant Enterobacterales (CRE), extensively drug-resistant (XDR) *Acinetobacter baumannii*, and multidrug-resistant (MDR) *Salmonella Typhi*, representing WHO priority organisms relevant to both clinical care and surveillance.

### Pathogens and AMR genes included:

The simulation incorporated AMR determinants commonly circulating in Pakistan, including beta-lactamases (*blaCTX-M*, *blaTEM*, *blaSHV*, *blaNDM-1*, *blaOXA-48*), methicillin resistance genes (*mecA*, *mecC*), aminoglycoside resistance genes (*aac(6’)-Ib*), fluoroquinolone resistance determinants (*qnrB*, mutations in *gyrA* and *parC*), colistin resistance (*mcr-1*), and carbapenemases (*blaKPC*, *blaVIM*, *blaIMP*).

### WHO Workflow Parameters:

Key WHO-recommended sequencing workflow parameters were applied, including minimum 30× genome coverage, base quality of Q30 or higher, mean read lengths ≥300 bp for short reads or ≥10 kb for long reads, a minimum metadata set (organism, specimen type, AST profile, collection date, hospital ward, patient age), contamination thresholds <5%, and assembly N50 >40 kb (9). The simulation framework assessed AMR gene detection accuracy, virulence gene identification, plasmid and mobile genetic element (MGE) reconstruction, SNP-based genotyping resolution, expected turnaround time, and practical feasibility in Pakistan, benchmarking outputs using published concordance metrics.^19^,^20^

## RESULTS

### AMR Gene detection:

Whole-genome sequencing (WGS) demonstrated exceptionally high predicted accuracy for detecting antimicrobial resistance (AMR) genes across the simulated priority pathogens. When evaluated against WHO-recommended quality standards including minimum 30× genome coverage, high-fidelity base calling, and stringent contamination thresholds WGS achieved 96–100% detection of key beta-lactamase genes responsible for resistance to cephalosporins and carbapenems in Pakistan, including *blaNDM-1*, the predominant carbapenemase associated with Enterobacterales outbreaks; *blaOXA-48*, frequently detected in *Klebsiella pneumoniae* and *Escherichia coli* from tertiary-care centers^3^; and *blaCTX-M*, particularly the CTX-M-15 variant, which dominates in South Asia. Simulation confirmed that these beta-lactamase genes, whether plasmid-borne or chromosomal, are consistently and accurately identified due to their conserved sequences, high expression, and stable genomic loci. Methicillin resistance genes in *Staphylococcus aureus*, including *mecA* and the less common *mecC*, were detected with ≥98% accuracy. WGS also showed >95% sensitivity for chromosomal AMR-associated mutations, such as *gyrA* S83L and *parC* S80I in *K. pneumoniae* and *E. coli*, and *gyrA*/*gyrB* mutations associated with quinolone-resistant *Salmonella Typhi*. Collectively, these results (Table-I) demonstrate that WGS can simultaneously identify plasmid-encoded resistance genes, chromosomal mutations, hybrid AMR loci, and rare variants, making it uniquely suited for surveillance in Pakistan, where AMR is genetically diverse and frequently mediated by mobile genetic elements.

**Table-I:** AMR genes detection by WGS.

S.No	Beta-lactamases encoding genes			
	Pathogens	Genes/Mutations	Detection Accuracy	Implication
1	Enterobacterales	blaNDM-1	96–100%	Predominant carbapenemase in outbreaks in Pakistan
2	Klebsiella pneumoniae, Escherichia coli	blaOXA-48	96–100%	Frequently detected in tertiary-care centers
3	Multiple Enterobacterales	blaCTX-M (esp. CTX-M-15)	96–100%	Dominant in South Asia; plasmid-borne or chromosomal
***Methicillin resistance genes***				
1	Staphylococcus aureus	mecA, mecC	>95%	
***Chromosomal mutations***				
1	K. pneumoniae, E. coli	gyrA S83L, parC S80I	>95%	Associated with quinolone resistance
2	Salmonella Typhi	gyrA/gyrB mutations	>95%	Linked to quinolone resistance

### Virulence Gene Identification:

In addition to AMR profiling, WGS enabled comprehensive identification of virulence factors that contribute to pathogenicity, transmissibility, and outbreak severity. Simulation showed ≥92% detection accuracy for MRSA virulence determinants, including Panton-Valentine leukocidin (PVL) genes (*lukF-PV*, *lukS-PV*), hemolysins (*hla, hlb, hld*), and enterotoxins (*sea, seb, sec*), which are associated with invasive community-acquired infections and immune evasion. For *K. pneumoniae*, WGS detected hypervirulence and capsule-associated loci with ≥95% accuracy, including capsular polysaccharide synthesis genes (K1, K2, K5, K57), siderophore systems (yersiniabactin *ybt*, aerobactin *iuc*, enterobactin *ent*), and mucoid phenotype regulators (*rmpA, rmpA2*) summarized in Table-II. Simultaneous detection of AMR and virulence genes facilitates real-time risk assessment and outbreak management, which is particularly valuable in Pakistani hospital settings.

**Table-II:** Virulence Gene Detection by WGS.

S.no	Virulence determinants			
	Pathogens	Genes/Loci	Detection Accuracy	
1	Staphylococcus aureus (MRSA)	PVL genes (lukF-PV, lukS-PV)	≥92%	Associated with invasive community-acquired infections and immune evasion
2	Staphylococcus aureus	Hemolysins (hla, hlb, hld)	≥92%	Contribute to host cell lysis and tissue damage
3	Staphylococcus aureus	Enterotoxins (sea, seb, sec)	≥92%	Linked to toxin-mediated disease and outbreak severity
4	Klebsiella pneumoniae	Capsular polysaccharide synthesis genes (K1, K2, K5, K57)	≥95%	Capsule types associated with hypervirulence and immune evasion
5	Klebsiella pneumoniae	Siderophore systems (yersiniabactin *ybt*, aerobactin *iuc,* enterobactin *ent*)	>95%	Enhance iron acquisition, critical for pathogenicity
6	Klebsiella pneumoniae	Mucoid phenotype regulators (rmpA, rmpA2	>95%	Promote hypermucoviscosity phenotype, linked to severe infections

### Plasmid and Mobile Genetic Element Reconstruction:

WGS also enabled precise reconstruction of plasmids and mobile genetic elements (MGEs), critical drivers of AMR dissemination in low- and middle-income countries, including Pakistan. Simulated workflows reconstructed plasmids carrying clinically important resistance genes such as *blaNDM-1* on IncA/C2, IncFII, and IncX3 plasmids; *blaOXA-48* on IncL/M plasmids; and *mcr-1* conferring plasmid-mediated colistin resistance. Accurate plasmid reconstruction allows differentiation between clonal and horizontal transmission, identification of plasmid types, and tracking of hospital- or community-level dissemination. WGS also detected a wide array of MGEs, including insertion sequences (ISAba, ISCR1), class-1 and class-2 integrons carrying multi-gene cassettes, and transposons such as Tn125 associated with *blaNDM-1* mobilization. The ability to reconstruct plasmids and MGEs supports infection control measures and stewardship programs by revealing the genetic mechanisms driving AMR outbreaks.

Simulation demonstrated excellent discrimination among MRSA lineages (CC8, CC5, CC22), classification of high-risk ESBL-*K. pneumoniae* clones (ST11, ST15, ST147), CRE lineages (ST167, ST410, ST405), and XDR *A. baumannii* global clone 2 (GC2). Transmission clusters were reliably detected using SNP distances of <10, enabling rapid identification of nosocomial transmission, silent carriage, and linking of cases to wards or medical devices. Simulated WGS workflows indicated that AMR gene detection could be achieved within 6–12 hours after obtaining a pure colony, representing a major improvement over traditional culture-based antimicrobial susceptibility testing, which typically requires 48–72 hours. This rapid turnaround, combined with comprehensive detection of resistance and virulence determinants, plasmid reconstruction, and high-resolution typing, underscores WGS as a transformative tool for AMR surveillance and outbreak management in Pakistan.

## DISCUSSION

The present study demonstrates that whole-genome sequencing (WGS), when applied in accordance with WHO technical guidelines, has substantial potential to transform antimicrobial resistance (AMR) detection and surveillance in Pakistan.

The WHO emphasizes WGS as a core component of modern AMR surveillance, recommending its integration into national programs, particularly for low- and middle-income countries (LMICs) that aim to detect emerging resistance threats early and implement evidence-based interventions.^21^ Several global studies demonstrate that WGS-based AMR surveillance facilitates early identification of novel resistance genes, tracking of plasmid-mediated transmission, and rapid detection of high-risk clones, thereby supporting antimicrobial stewardship programs and guiding treatment strategies.

In this study WGS achieved detection of beta-lactamase genes including *blaNDM-1*; the predominant carbapenemase associated with Enterobacterales outbreaks, *blaOXA-48*; frequently detected in *Klebsiella pneumoniae* and *Escherichia coli* from tertiary-care centers^22^ and *blaCTX-M*, particularly the CTX-M-15 variant; which dominates in South Asia, with exceptional accuracy (96–100%). Simulation confirmed that these beta-lactamase genes, whether plasmid-borne or chromosomal, are consistently and accurately identified due to their conserved sequences, high expression, and stable genomic loci that underpin the reliability of WGS for monitoring carbapenem and cephalosporin resistance.

Methicillin resistance genes (*mecA* and *mecC)* in *Staphylococcus aureus* were detected with ≥98% accuracy. Chromosomal AMR-associated mutations in *K. pneumoniae* and *E. coli* (*gyrA* S83L and *parC* S80I) and quinolone-resistant *Salmonella Typhi* (*gyrA*/*gyrB)* were detected with >95% accuracy. These observations suggest WGS as a comprehensive tool for the detection of chromosomal as well as plasmid-mediated mechanisms of resistance.

Beyond AMR profiling, virulence determinants were also identified with remarkable accuracy (≥92–95%) via WGS. MRSA virulence determinants, including Panton-Valentine leukocidin (PVL) genes (*lukF-PV*, *lukS-PV*), hemolysins (*hla, hlb, hld*), and enterotoxins (*sea, seb, sec*), associated with invasive community-acquired infections and immune evasion, were determined with ≥92% accuracy. In *K. pneumoniae*, hypervirulence loci including capsular polysaccharide synthesis genes (K1, K2, K5, K57), siderophore systems (yersiniabactin, aerobactin, enterobactin), and mucoid phenotype regulators (rmpA, rmpA2) were reconstructed with high fidelity. This dual profiling of resistance and virulence is particularly valuable for real-time risk assessment, as it allows clinicians and public health authorities to anticipate both therapeutic challenges and outbreak severity.

WGS also enabled precise reconstruction of plasmids and mobile genetic elements (MGEs), critical drivers of AMR dissemination in low- and middle-income countries, including Pakistan. Simulated workflows reconstructed plasmids carrying clinically important resistance genes such as *blaNDM-1* on IncA/C2, IncFII, and IncX3 plasmids; *blaOXA-48* on IncL/M plasmids; and *mcr-1* conferring plasmid-mediated colistin resistance. Accurate plasmid reconstruction allows differentiation between clonal and horizontal transmission, identification of plasmid types, and tracking of hospital- or community-level dissemination. WGS also detected a wide array of MGEs, including insertion sequences (ISAba, ISCR1),), class-1 and class-2 integrons carrying multi-gene cassettes, and transposons such as Tn125 associated with *blaNDM-1* mobilization. The ability to reconstruct plasmids and MGEs supports infection control measures and stewardship programs by revealing the genetic mechanisms driving AMR outbreaks. The reliability of WGS workflows for detecting methicillin-resistant *Staphylococcus aureus* (MRSA) and ESBL-producing *Klebsiella pneumoniae*, as demonstrated by literature^14^ further corroborates the validity of our simulation outputs in the context of Pakistan.

This is the first simulation-based study validating WHO-recommended WGS workflows for AMR detection in Pakistan. Findings of this study suggest that WGS can achieve remarkable detection of both plasmid-borne and chromosomal resistance determinants, as well as virulence loci and mobile genetic elements via modelling realistic genomic parameters and pathogens. Moreover, WGS provides rapid turnaround and comprehensive profiling, unlike conventional Antimicrobial susceptibility testing. This study adapts global guidelines for WGS-based AMR detection in Pakistan, thereby bridging the gap in the medical literature on genomic AMR surveillance in low- and middle-income countries.

Our findings underscore WGS as a transformative tool for patient care and public health in Pakistan due to its underlying ability to detect resistance and virulence determinants simultaneously within 6–12 hours. This fast profiling provides prompt infection control measures, selection of the appropriate antimicrobial therapy, and prediction of outbreak severity.

The integration of WHO technical standards, ensuring global comparability and methodological rigor, highlights the strength of these findings. Furthermore, the contextual relevance of this study is underpinned by the incorporation of leading pathogens involved in Pakistan’s AMR. Feasibility within tertiary-care settings provides actionable insights for implementation across Pakistan. Additionally, Integration of WGS into national AMR surveillance systems will scale up reporting and outbreak management.

### Limitations:

The primary limitation of the study is the simulation-based design. While the simulation was based on realistic genomic parameters, practical implementation of WGS in Pakistan faces practical challenges, including limited bioinformatics expertise, infrastructure constraints, inconsistent power supply, and difficulties in importing reagents. However, the country’s tertiary-care hospitals and provincial public health laboratories possess the foundational equipment and trained personnel to initiate WGS workflows, suggesting that phased implementation is feasible.^23^,^24^ Incorporating WGS into the national AMR action plan can close the existing surveillance gap identified by the Ministry of National Health Services, enabling evidence-driven public health interventions and integration with global reporting platforms such as WHO GLASS.^25^

## CONCLUSION

Whole-genome sequencing (WGS) is a practical, accurate, and very useful tool for detecting antimicrobial resistance (AMR) in Pakistan. Our simulation shows that the WHO-recommended WGS workflow can be used in most tertiary-care laboratories with the facilities they already have. However, to successfully adopt WGS across the country, Pakistan needs to invest in basic bioinformatics training, proper quality-control systems, and better connections between laboratories. These steps will help ensure reliable AMR detection and support a strong national surveillance system.

### Recommendations:

Further research should validate the simulation-based findings under real-world conditions in Pakistan. Long-term sustainability in resource-limited settings underscores Cost-effectiveness studies.
